# Bioremediation potential of consortium *Pseudomonas Stutzeri* LBR and *Cupriavidus Metallidurans* LBJ in soil polluted by lead

**DOI:** 10.1371/journal.pone.0284120

**Published:** 2023-06-15

**Authors:** Sirine Ridene, Naima Werfelli, Ahlem Mansouri, Ahmed Landoulsi, Chiraz Abbes

**Affiliations:** 1 University of Carthage, Biochemistry and Molecular Biology Laboratory of Faculty of Sciences of Bizerte, Risks Related to Environmental Stress, Struggle and Prevention (UR17ES20), Bizerte, Zarzouna, Tunisia; 2 International Center For Environmental Technologies, Boulevard Leader Yasser Arafat, Tunis, Tunisia; Universidad Tecnica de Manabi, ECUADOR

## Abstract

Pollution by lead (Pb) is an environmental and health threat due to the severity of its toxicity. Microbial bioremediation is an eco-friendly technique used to remediate contaminated soils. This present study was used to evaluate the effect of two bacterial strains isolated and identified from Bizerte lagoon: *Cupriavidus metallidurans* LBJ (*C*. *metallidurans* LBJ) and *Pseudomonas stutzeri* LBR (*P*. *stutzeri* LBR) on the rate of depollution of soil contaminated with Pb from Tunisia. To determine this effect, sterile and non-sterile soil was bioaugmented by *P*. *stutzeri* LBR and *C*. *metallidurans* LBJ strains individually and in a mixture for 25 days at 30°C. Results showed that the bioaugmentation of the non-sterile soil by the mixture of *P*. *stutzeri* LBR and *C*. *metallidurans* LBJ strains gave the best rate of reduction of Pb of 71.02%, compared to a rate of 58.07% and 46.47% respectively for bioaugmentation by the bacterial strains individually. In the case of the sterile soil, results showed that the reduction rate of lead was in the order of 66.96% in the case of the mixture of the two bacterial strains compared with 55.66% and 41.86% respectively for the addition of the two strains individually. These results are confirmed by analysis of the leachate from the sterile and non-sterile soil which showed an increase in the mobility and bioavailability of Pb in soil. These promising results offer another perspective for a soil bioremediation bioprocess applying bacterial bioremediation.

## Introduction

Over recent decades, the mining industry has played an overwhelming role in global energy and has generated significant economic benefits annually for several regions of the country [1]. However, mining is one of the industrial activities that causes the largest and most persistent alterations to the ecosystem and human health [2], including loss of biodiversity and accumulation of pollutants in the environment. It represents one of the main sources of heavy metals and metalloids [3] pollution in the soil, due to the large quantities of discharges with high levels of toxic chemicals [4] and stocks that are often abandoned without treatment [3]. This can lead to intense destruction of natural environments [5] through soil and water pollution due to high concentrations of heavy metals in mine waste [1]. Heavy metals are defined as trace metal elements (TMEs) with an atomic number greater than 20 and an elemental density greater than 5 g/cm^3^ [6]. Heavy metals are highly toxic, non-biodegradable [7], persistent in ecosystems [3] and are characterized by their ability to bioaccumulate in food chains [8]. Heavy metals can be grouped into essential and non-essential classes [9]. Essential heavy metals including manganese (Mn), magnesium (Mg), nickel (Ni), iron (Fe), zinc (Zn) and copper (Cu) are considered as non-toxic micronutrients, which are involved in the maintenance of various biochemical and physiological functions and are present at low concentrations in living organisms [4]. However, they can become toxic when they are taken in excessive quantities that exceed the threshold [9]. Non-essential heavy metals including Silver (Ag), Cadmium (Cd), Arsenic (As), Mercury (Hg) and Lead (Pb) are highly toxic to living organisms even at low concentrations [4]. Soil contamination by heavy metals has become a major concern for scientists because of their toxicities, which pose a major threat to the environment [10]. In fact, the uncontrolled release of heavy metals into the soil and waterways implies adverse effects on fauna and flora [8]. In particular, lead (Pb) raises a serious concern for scientists due to its high toxicity and long-term persistence in the environment [11]. It is also characterized by its ability to accumulate in plants and human organisms causing serious irreversible damage [12]. This damage can lead to anaemia, reproductive disorders, kidney failure, neurodegenerative damage and cardiovascular disease [11]. Therefore, soil pollution with high concentrations of Pb from mining is one of the most severe environmental problems that degrade ecosystems and disrupt natural biodiversity [2,13]. The need to adapt specific depollution strategies becomes necessary. Recently, various physicochemical techniques such as chemical leaching [14], membrane filtration, stabilization/solidification [15], reverse osmosis, evaporation, electrochemical treatment, ion exchange, sorption and precipitation have been applied to remediate contaminated sites and remove contaminants, including heavy metals [11,12]. However, they present numerous limitations, including the production of toxic chemical sludge that is not eco-friendly [2]. Nowadays, a series of bioremediation technologies have attracted the attention of scientists because of their exceptional advantages and their high efficiency in cleansing soils contaminated by heavy metals [2]. Especially, microbial bioremediation is envisioned as a biological treatment technique for polluted soil [16], applied for the removal and/or recovery of TMEs in contaminated environments through the use of microorganisms [3]. It is an efficient, promising and environmentally friendly depollution technique [17] compared to physicochemical techniques. A wide variety of microorganisms (fungi, algae, bacteria) are used in the bioremediation process [2] as biological tools to rehabilitate environments polluted by heavy metals through their development of resistance mechanisms such as bioaccumulation, biomineralization, biosorption and biotransformation [3] in order to adapt to the different toxic metals in ecosystems. Various bacteria have the capacity to remove pollutants from the environment and are used in remediation methods such as the bacterial strains *Pseudomonas stutzeri* LBR (*P*. *stutzeri* LBR) and *Cupriavidus metallidurans* LBJ (*C*. *metallidurans* LBJ), selected and identified in our laboratory from Tunisian sediment and which are characterized by their ability to interact with multiple contaminants as sources of carbon and energy [18]. The *P*. *stutzeri* LBR strain is used in bioremediation technology in environments affected by heavy metals. It is considered cosmopolitan and it presents a range of biochemical and physiological traits such as nitrogen fixation, denitrification and metal biosorption [19,20]. Moreover, *P*. *stutzeri* LBR contributes to the degradation of pollutants and interacts with toxic metals [21]. *C*. *metallidurans* LBJ is a model strain resistant to heavy metals that can support millimolar concentrations of heavy metals. It develops genes encoding mega-plasmids that specify heavy metal resistance functions [18]. *C*. *metallidurans* LBJ has been applied for bioremediation, recovery and limitation of heavy metals [18]. It carried many determinants of heavy metal resistance implicated in different mechanisms, including efflux systems, complexation, reduction, and reductive precipitation, which enable cell detoxification and survival [22]. In our study, we used the bioaugmentation process, which consists of the addition of pre-cultured microbial cultures (individually or in consortium) *P*. *stutzeri* LBR and *C*. *metallidurans* LBJ, with a high power to degrade pesticides, hydrocarbons and heavy metals, in order to improve the degradation of pollutants. This process is one of the most common and best-suited approaches for bioremediation of polluted sites and remediation of in situ contaminants with minimal impact on surface ecosystems [23]. Compared to ex-situ bioremediation techniques such as pumping, biopile and primary treatment, bioaugmentation is a low-cost, environmentally friendly method that generates little waste after treatment. The main objectives of this research were:

- To study the effectiveness of bacteria *P*. *stutzeri* LBR and *C*. *metallidurans* LBJ in remediating Pb-contaminated soil through the use of the microbial bioremediation process of bioaugmented bacterial strains individually and in consortium,
- To follow the rate of reduction of Pb concentration as a function of bacterial growth kinetics while analyzing the concentration of Pb in the leachate (the medium in which the soil bathes throughout the bioremediation experiment).

## Materials and methods

### Study area and soil samples

Soil samples were taken from a highly polluted area with heavy metals; it is a mining area located in Tunisia. Samples were collected from 0 to 20 cm below the soil surface [10], by quincunx sampling method, then they were sieved (mesh size two mm), mixed, and transported to the laboratory in sterile bottles at 4°C, in accordance with the ISO 19458 sampling standard.

### Physicochemical characteristics

The physicochemical characteristics of the soil sample, such as pH, electrical conductivity, salinity, dry matter (DM) and humidity (H), were measured in accordance with AFNOR NF ISO-10390, November (1994), NF ISO 11265, January (1995) and NF ISO 114465 (1994) respectively.

### Dosage of heavy metals

The dosage of heavy metals in the soil sample was determined using Inductively Coupled Plasma Optical Emission Spectrophotometry ICP-OES (PerkinElmer Optima 7300 DV, Inc., Shelton, CT, USA) in accordance with the requirements of ISO 11885, March 1998.

### Bacterial strains used in bioremediation by bioaugmentation of soil contaminated with heavy metals

The bacterial strains used in bioremediation by bioaugmentation of soil contaminated with Pb are: *P*. *stutzeri* LBR strain and *C*. *metallidurans* LBJ strain, isolated from sediment of Bizerte lagoon in our Laboratory of Biochemical and Molecular Biology in the Faculty of Sciences of Bizerte, Tunisia in the work of Mansouri and colleagues [18]. The sequences of these bacterial strains *P*. *stutzeri* LBR and *C*. *metallidurans* LBJ have been deposited in the GenBank database [18]. These isolated bacterial strains were transplanted to the PCA culture medium and incubated at 37°C for 24–48 hours. After incubation, these strains were maintained on glycerol at -80°C for long-term conservation. The morphological characterization and Gram staining of the bacterial strains *P*. *stutzeri* LBR and *C*. *metallidurans* LBJ proves that both strains are bacilli Gram (-), of Star shape with irregular contour (umbilical) and pink colour for the strain *P*. *stutzeri* LBR, and of regular Circular shape of White contour with Yellow point for the strain *C*. *metallidurans* LBJ. In terms of biochemical characterization, the strain *P*. *stutzeri* LBR gave positive reaction for oxidase activity, coagulase activity, fermentation of glucose, lactose and negative reaction for catalase activity, H_2_S, Gas, and mannitol. The *C*. *metallidurans* LBJ strain characterized by a positive reaction for catalase activity, fermentation of glucose, lactose, H_2_S^+^, Gas^+^ and a negative reaction for oxidase activity, coagulase activity and mannitol [18].

### Bioaugmentation tests of contaminated soil sample

For bioaugmentation tests in the sterile and non-sterile soil, we followed the same steps except, in the case of the sterile soil, sterilizing it by autoclaving at 120°C for 15 min [16]. In a flask, 10 g of soil was mixed with 500 mL of sterilized M9 medium, then 70 x 10^9^ bacteria cells were inoculated. The bioaugmentation experiments were carried out in four flasks for both sterile and non-sterile soil. The different compositions used in the experiment on bioaugmentation in the sterile soil were: (i) (Pb SS1): Sterile soil + M9 (glycerol) + *P*. *stutzeri* LBR + *C*. *metallidurans* LBJ, (ii) (Pb SS2): Sterile soil + M9 (glycerol) + *P*. *stutzeri* LBR, (iii) (Pb SS3): Sterile soil + M9 (glycerol) + *C*. *metallidurans* LBJ, (iv) (C. SS): Sterile soil + M9 (glycerol). The different compositions used in the experiment on bioaugmentation in the non-sterile soil were: (i) (Pb NS1): Non-sterile soil + M9 (glycerol) + *P*. *stutzeri* LBR + *C*. *metallidurans* LBJ, (ii) (Pb NS2): Non-sterile soil + M9 (glycerol) + *P*. *stutzeri* LBR, (iii) (Pb NS3): Non-sterile soil + M9 (glycerol) + *C*. *metallidurans* LBJ, (iv) (C. NS): Non-sterile soil + M9 (glycerol). All of the flasks were stirred at 30°C under aerobic conditions and under agitation (100 rpm), for 25 days [24]. All treatments were performed in triplicate. Every five days the bacterial counts and analysis of Pb by ICP-OES was realized. All necessary aseptic conditions were respected.

### Statistical analysis

Means and standard deviations were calculated using STATISTICA 8.0. All analyses were performed three times. The comparative analysis of the data was determined with an analysis of variance test (ANOVA) with significant differences at p<0.05. R Foundation for Statistical Computing Version 3.6.3 statistical software was used for testing (R Development Core Team, 2009–2018 RStudio, Inc.).

## Results

### Study of the physicochemical parameters of the soil sample

The results for physicochemical characteristics showed that the pH measured in soil showed an acidic pH of about 5.09 at a temperature of 17.70°C. This result promotes the trophic transfer of heavy metals [25].

The electrical conductivity of the polluted soil shows a value of 603.06 μS/cm. This value can describe a very low salinity of the order of 0.22. Another value, the percentage of dry matter and soil humidity, shows that it is a soil rich in dry matter (96% DM) and low humidity 2%.

### Dosage of heavy metals in soil sample

The analysis of the metal elements in the polluted soil sample by ICP shows a significant increase in the concentrations of heavy metals in the soil compared to the standard set by the European Directive. In particular, Pb has the highest concentration, which is in the order of 896.84 ± 0.04, three times higher than the standard of the European Directive (50 to 300). This means that the soil is highly saturated with lead. Cadmium also exceeds the European standard set with a concentration of 18.84 ± 0.03. The other values do not present a serious environmental problem as shown in Table 1.

**Table 1:** Concentration of heavy metals in polluted soil (mg/kg DM).

Heavy metals	Unity	Concentration	Limit values according to the European Directive
Cadmium	mg/kg DM	18.84 ± 0.03	1 to 3
Cobalt		14.86 ± 0.02	-
Copper		14.42 ± 0.01	50 to 140
Iron		242.56 ± 0.02	-
**Lead**		**896.84 ± 0.04**	**50 to 300**
Manganese		98.34 ± 0.01	-
Nickel		15.8 ± 0.02	30 to 75
Zinc		212.92 ± 0.01	150 to 300
Chromium		22.47 ± 0.007	-

### Soil remediation study by microbial bioremediation in sterile soil

**Fig 1:**
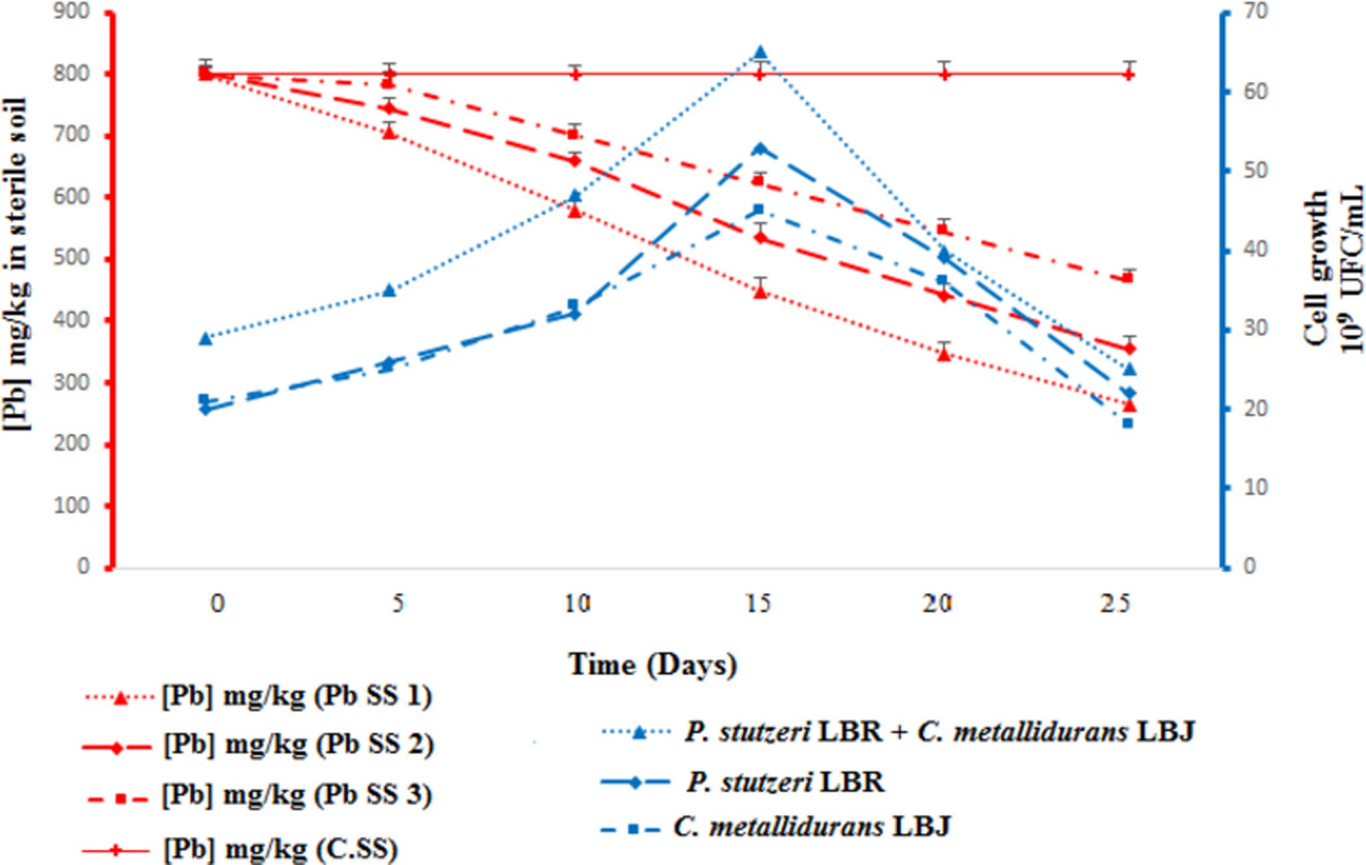
Bioaugmentation of polluted sterile soil by *P*. *stutzeri* LBR and *C*. *metallidurans* LBJ. (Pb SS 1) Polluted sterile soil bioaugmented by the consortium of *P*. *stutzeri* LBR and *C*. *metallidurans* LBJ. (Pb SS 2) Polluted sterile soil bioaugmented by *P*. *stutzeri* LBR strain. (Pb SS 3) Polluted sterile soil bioaugmented by *C*. *metallidurans* LBJ strain. (C.SS) Control sterile soil (not inoculated with the strain).

The result of bioaugmentation of sterile soil by the bacterial strains *P*. *stutzeri* LBR and *C*. *metallidurans* LBJ are presented in Fig 1. To monitor the rate of Pb reduction, we have tracked the growth kinetics of these bacterial strains over 25 days at 30°C.

In the first 15 days of the experiment (phase 1), we observed that the increase in the bacterial growth rate of the strains *P*. *stutzeri* LBR and *C*. *metallidurans* LBJ in consortium led to a significant reduction (p< 0.05) of Pb concentration in sterile soil.

Under these conditions, the Pb concentration fell from 800.05 mg/kg to 264.41 mg/kg within 25 days at 30°C, which corresponds to a percentage reduction of 66.96%. During this phase, the number of bacteria in the consortium reached a peak of 65.109 CFU/mL, followed by a decrease in the number of cells, which reached 25.109 CFU/mL at the end of the experiment.

In the sterile soil bioaugmented with the *P*. *stutzeri* LBR strain, we found that the total bioaugmented soil flora showed growth during the first 15 days (phase 1), followed by a decrease in the number of cells at the end of the experiment. This is accompanied by a significant reduction in the Pb concentration from 801.08 mg/kg to 355.18 mg/kg over 25 days, with a reduction percentage of 55.66%. The increase in bacterial growth rate of the individually bioincreased *C*. *metallidurans* LBJ strain during the first phase of the experiment resulted in a significant reduction in Pb concentration from 800.41 mg/kg to 465.37 mg/kg over 25 days, with a reduction percentage of 41.86%. Beyond this phase, the number of cells of the *C*. *metallidurans* LBJ strain decreased. Therefore, the sterile soil bioaugmented with the consortium showed a significant decrease in Pb concentration compared to the sterile soil bioaugmented with individual strains. These results are confirmed by the statistical analysis on the R software showing that there is a significant difference between the three Pb reduction rates obtained under the different conditions Pb SS1, Pb SS2 and Pb SS3.

### Leachate analysis after sterile soil bioaugmentation

**Fig 2:**
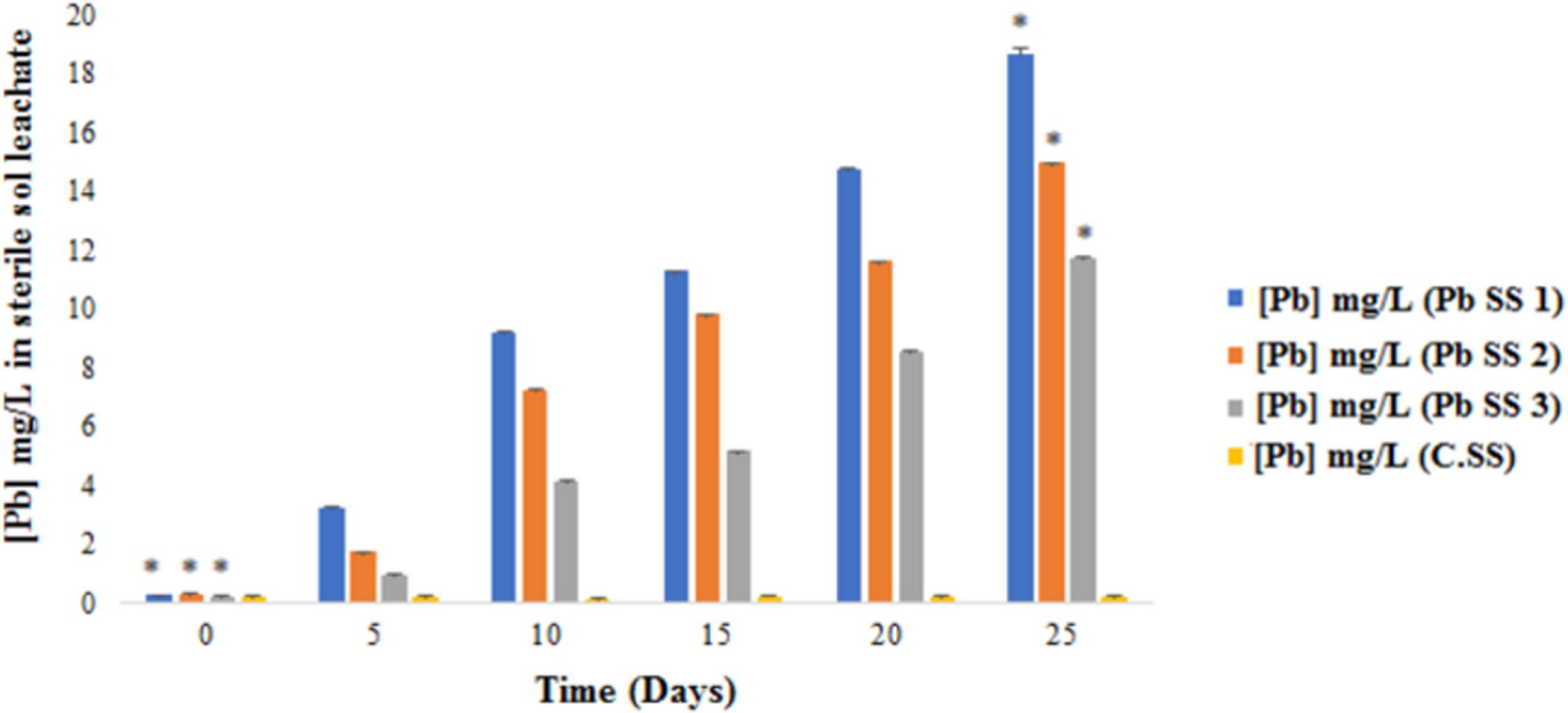
Evolution of the concentration of Pb in the leachate of bioaugmented sterile soil. (Pb SS 1) Polluted sterile soil bioaugmented by the consortium of *P*. *stutzeri* LBR and *C*. *metallidurans* LBJ. (Pb SS 2) Polluted sterile soil bioaugmented by *P*. *stutzeri* LBR strain. (Pb SS 3) Polluted sterile soil bioaugmented by *C*. *metallidurans* LBJ strain. (C.SS) Control sterile soil (not inoculated with the strain).

To verify the effect of the bacterial strains used on the reduction of the concentration of Pb in soil, an analysis of the leachate of the sterile soil (the medium in which the soil bathes throughout the experiment) was carried out throughout the bio augmentation experiment. Fig 2 represents the evolution of the concentration of Pb in the leachate of sterile soil bioaugmented with bacterial strains as a function of time.

This figure shows that the concentration of Pb in the leachate of sterile soil bioaugmented with bacterial strains increased significantly compared to the control (C.SS) over 25 days of the experiment. During this period, the concentration of Pb in leachate increased from 0.29 mg/L to 18.63 mg/L in soil bioaugmented with the consortium, from 0.33 mg/L to 14.96 mg/L in soil bioaugmented with *P*. *stutzeri* LBR, and from 0.22 mg/L to 11.76 mg/L in soil bioaugmented with the *C*. *metallidurans* LBJ strain.

Therefore, there is a significant difference (p<0.05) between sterile soil bioaugmented with the bacterial consortium and individually bioaugmented soil compared to the control.

### Soil remediation study by microbial bioremediation in non-sterile soil

**Fig 3:**
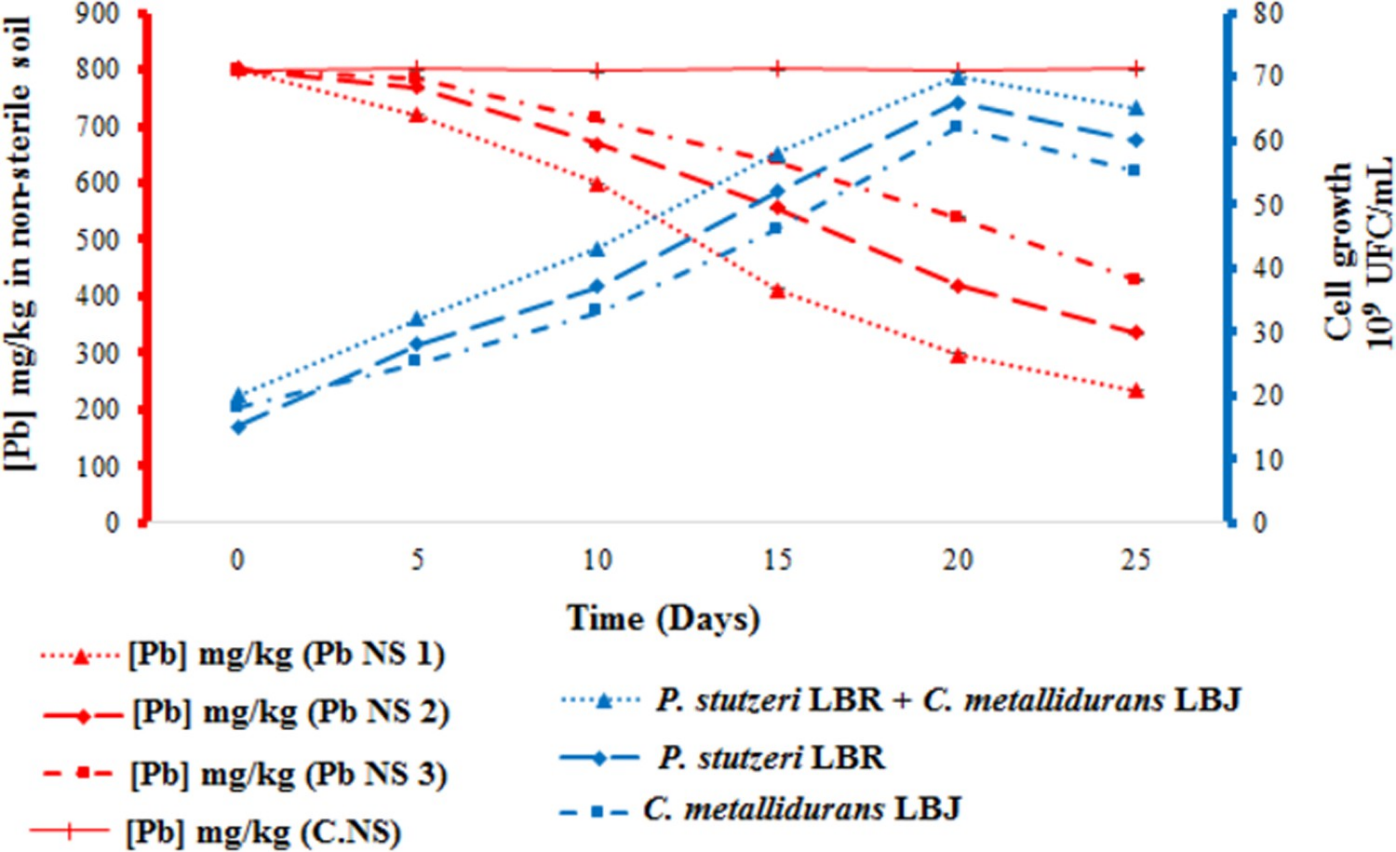
Bioaugmentation of polluted non-sterile soil with *P*. *stutzeri* LBR and *C*. *metallidurans* LBJ. (Pb NS 1) Polluted non-sterile soil bioaugmented with the consortium of *P*. *stutzeri* LBR and *C*. *metallidurans* LBJ. (Pb NS 2) Polluted non-sterile soil bioaugmented with the *P*. *stutzeri* LBR strain. (Pb NS 3) Polluted non-sterile soil bioaugmented by the *C*. *metallidurans* LBJ strain. (C.NS) Control non-sterile soil (not inoculated with the strain).

Another experiment in the bioaugmentation of non-sterile soil with the bacterial strains *P*. *stutzeri* LBR and *C*. *metallidurans* LBJ was realized to track the rate of Pb reduction as a function of bacterial growth over 25 days at 30°C (Fig 3).

This figure shows that the growth rate of the entire bacterial population in the presence of *P*. *stutzeri* LBR and *C*. *metallidurans* LBJ strains in consortium in non-sterile soil increased during the first 20 days of the experiment (phase 1) from 20.109 CFU/mL to 70.109 CFU/mL. This increase resulted in a significant reduction (p<0.05) in Pb concentration from 801.33 mg/kg to 232.19 mg/kg over 25 days at 30°C, with a percentage reduction of 71.02%. Beyond this phase, the total bacterial count decreased. The increase in growth rate of the entire bacterial population with the individually bioaugmented *P*. *stutzeri* LBR strain in the non-sterile soil during Phase 1 resulted in a significant reduction in Pb concentration. Under these conditions, the Pb concentration decreased from 801.28 mg/kg to 335.91 mg/kg over 25 days, corresponding to a reduction of 58.07%. In non-sterile soil bioaugmented with strain *C*. *metallidurans* LBJ, the number of the whole bacterial population of the bioaugmented soil showed an increase during the first 20 days (phase 1), followed by a decrease in the total number of bacterial cells at the end of the experiment. This is accompanied by a significant reduction in the Pb concentration from 799.74 mg/kg to 428.07 mg/kg over 25 days, with a percentage reduction of around 46.47%. Therefore, the bioaugmented non-sterile soil with the consortium showed a significant decrease in Pb concentration compared to the individually bioaugmented soil with the *P*. *stutzeri* LBR and *C*. *metallidurans* LBJ strains. These results are confirmed also by the statistical analysis showing that there is a significant difference between the three Pb reduction rates obtained under the different conditions Pb NS 1, Pb NS 2 and Pb NS 3.

### Leachate analysis after non-sterile soil bioaugmentation

**Fig 4:**
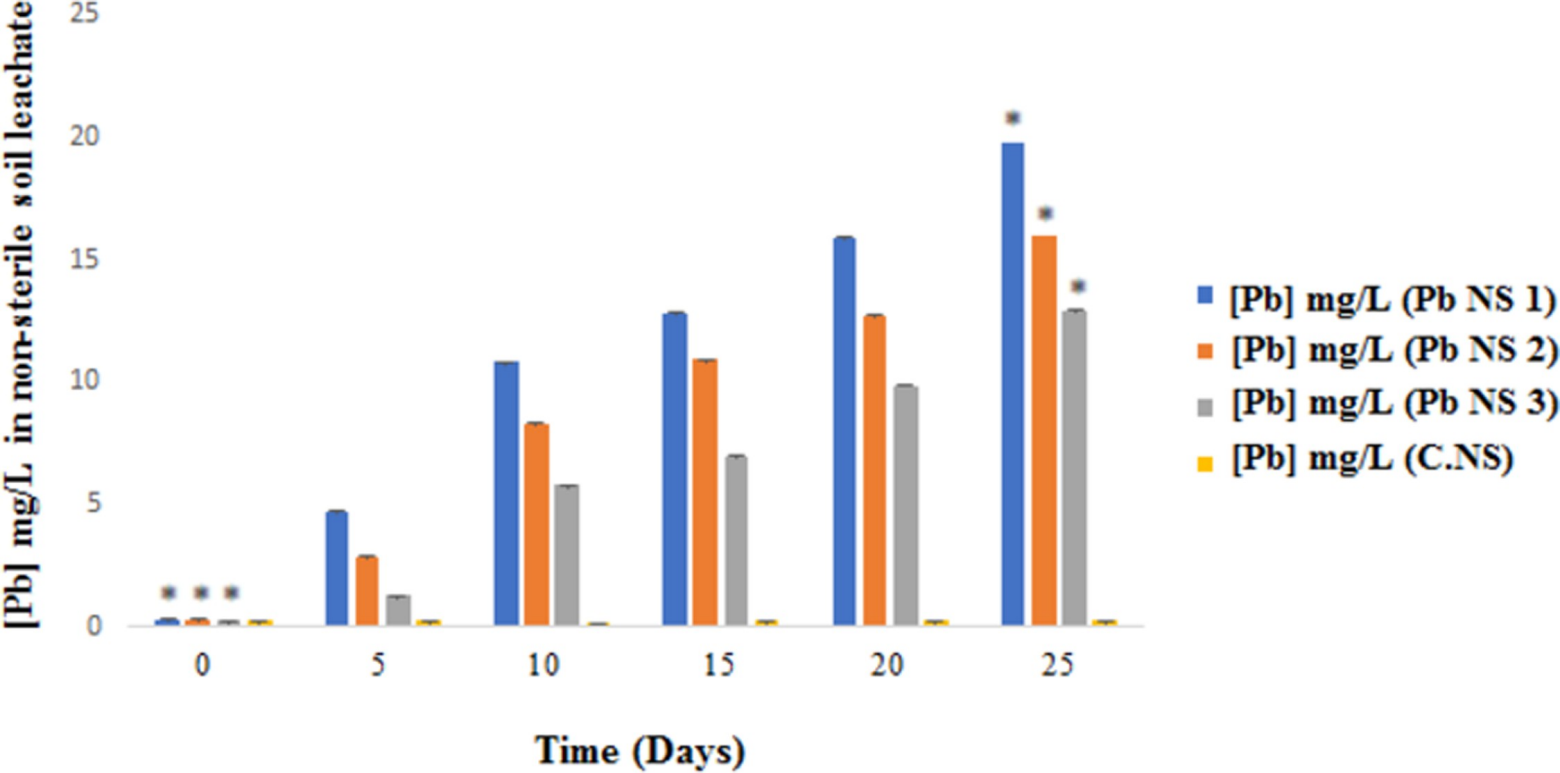
Evolution of the concentration of Pb in the leachate of bioaugmented non-sterile soil. (Pb NS 1) Polluted non-sterile soil bioaugmented with the consortium of *P*. *stutzeri* LBR and *C*. *metallidurans* LBJ. (Pb NS 2) Polluted non-sterile soil bioaugmented with the *P*. *stutzeri* LBR strain. (Pb NS 3) Polluted non-sterile soil bioaugmented with the *C*. *metallidurans* LBJ strain. (C.NS) Control non-sterile soil (not inoculated with the strain).

Similar to the sterile soil, a leachate analysis of the non-sterile soil (the environment in which the soil bathes throughout the experiment) was conducted throughout the bioaugmentation experiment to explain the effect of the entire bacterial population of non-sterile soil with the *P*. *stutzeri* LBR and *C*. *metallidurans* LBJ strains used, on the reduction of Pb concentration. The change in the concentration of Pb in the leachate of non-sterile soil bioaugmented with bacterial strains over time is shown in Fig 4.

From this figure we noted that during the 25 days of the experiment, the concentration of Pb in the leachate of the non-sterile soil bioaugmented with the bacterial strains increased significantly compared to the control (C.NS). Indeed, the concentration of Pb increased from 0.29 mg/L to 19.73 mg/L in the leachate of the bioaugmented soil with the consortium, from 0.28 mg/L to 15.98 mg/L in the soil bioaugmented with the *P*. *stutzeri* LBR strain, and from 0.25 mg/L to 12.92 mg/L in the soil bioaugmented with the *C*. *metallidurans* LBJ strain throughout the experimental period. Therefore, there is a significant difference (p<0.05) between the non-sterile soil bioaugmented with the bacterial consortium and the individually bioaugmented soil compared to the control.

## Discussion

This study was based on the microbial bioremediation of sterile and non-sterile polluted soil, contaminated with heavy metals, in particular Pb. For soil bioremediation, we used two bacterial strains *P*. *Stutzeri* LBR and *C*. *metallidurans* LBJ [18]. Depollution efficiency is influenced by the resistance capacity of *P*. *Stutzeri* LBR and *C*. *metallidurans* LBJ bacteria to Pb through their ability to adsorb and accumulate metal ions [26].

This work began with a physicochemical characterization of the soil sample. The pH analysis of the polluted soil showed an acidic pH. This result is in agreement with the work of Serpaud and colleagues [27] that showed that Pb tends to predominate in an acid medium (pH = 5.6). Indeed, the biosorption capacity of Pb on the bacterial cell surface is strongly dependent on increasing pH from 2 to 6, with a maximum uptake capacity at pH 5 [28]. Moreover, Sharma and colleagues [42] showed that pH can have a significant effect in helping bacteria to mobilize certain metals at pH 6.2 to 8. A series of bioaugmentation experiments was carried out over 25 days in sterile and non-sterile soil.

These tests allowed us to study the evolution of the rate of reduction in Pb concentration according to the kinetics of bacterial growth. The results showed that the increase in the bacterial growth rate led to a reduction in the concentration of Pb in the soil especially during phase 1 of the experiment. Beyond this phase, the decrease in the number of bacterial cells in the sterile soil and non-sterile soil may be due to the depletion of the carbon source (glycerol) added to the medium. The best Pb concentration reduction rate was observed in non-sterile soil bioaugmented with the consortium of *P*. *Stutzeri* LBR and *C*. *metallidurans* LBJ strains, with a value of 71.02% compared to a rate of 66.96% in the case of sterile soil bioaugmented with the consortium. These results showed that the bacterial consortium of the strains *P*. *stutzeri* LBR and *C*. *metallidurans* LBJ are characterized by a high efficiency of lead biosorption compared to the work of Waite and colleagues [29], who proved that the strain *P*. *stutzeri* W228 presents significant results for the absorption of Pb going to concentrations up to 54.0 mg/kg. Further, the work of Ayangbenro and Babalola [30] found that the strain of *Bacillus cereus* NWUAB01 isolated from mining soil showed a lead removal efficiency of 69%.

The increase in the rate of Pb reduction in non-sterile soil compared to sterile soil is apparently due to the combined action between the microbial population already present in the soil and the bioaugmented bacteria. In addition, the combined action of the consortium formed by the bacterial strains *P*. *Stutzeri* LBR and *C*. *metallidurans* LBJ in both the sterile and non-sterile soil showed a synergistic effect which was manifested by a rate of Pb reduction greater than that obtained in the case of soil bioaugmented with bacterial strains individually.

This result is consistent with the work of Diels and colleagues [31], who showed that the combination of resistance to heavy metals improves the bioprecipitation capacity of metals. Therefore, bacterial mixtures are proven to be the most effective microbial bioremediation method, compared to single-strain culture methods, which could be due to higher bacterial cell density. This hypothesis is confirmed by the work of Kang and colleagues [32], who concluded that the isolation and mixing of native bacteria (*Viridibacillus arenosi*, *Sporosarcina soli*, Enterobacter cloacae and E. cloacae) in the bioremediation process lead to increased growth rates and higher resistance to heavy metals than individual cultivation methods.

*P*. *stutzeri* LBR has preferential biosorption to Pb, with inertization of more toxic metals (Pb) compared to less toxic metals (Cu) [21]. This conclusion explained the result that the greater rate of Pb reduction appeared in the sterile and non-sterile soil bioaugmented only with the *P*. *Stutzeri* LBR strain respectively of 55.66% and 58.07%.

As a result, bacterial cells invest in the production of exopolysaccharides (ESP) (involved in metal resistance mechanisms [33]) which facilitates the biosorption process of Pb by *P*. *stutzeri* LBR by the existence of ionizable functional groups, such as carboxyl (COOH), phosphate (RHPO_4_), hydroxyl (R-OH), sulfhydryl (R-SH), phenolic (R C_6_H_5_OH) and amine (R-NH_2_), that permit them to bind toxic heavy metals, particularly Pb [34,35]. Likewise, the enzymatic activities of the ESP contribute to the inertization and detoxification of Pb via precipitation and transformation processes at the level of the polymer mass, creating a biosorbent agent that is particularly ideal for bioremediation due to their involvement in the flocculation process and the binding of metal ions from solutions [33,36,37]. Also, the structure and composition of the ESP varied according to the phase of bacterial growth, where a greater decrease of lead concentration was observed especially during phase 1 of growth, in sterile and non-sterile soil, due to the high incorporation of acidic sugar in the ESP [38].

The low Pb reduction rate in the sterile and non-sterile soil bioaugmented only with the strain *C*. *metallidurans* LBJ (41.86% and 46.47% respectively) compared to the Pb rate reduction in the sterile and non-sterile soil bioaugmented with *P*. *stutzeri* LBR only is explained by the higher affinity of *C*. *metallidurans* LBJ to Cadmium compared to Pb and thereby an average capacity of biosorption to some other metals despite being a multi-resistant bacterial model [39]. However, this reduction is explained by the ability of Pb to accumulate in polyphosphate inclusions [40]. Indeed, *C*. *metallidurans* LBJ has the pbr*ABCD* operon which encodes the pbrD protein involved in resistance to Pb. This is an intracellular protein which allows the sequestration of Pb using a binding site rich in residues of cysteine, proline and serine, which reduces its toxic effects [41].

To better check the efficacy of the bacterial strains used *P*. *stutzeri* LBR and *C*. *metallidurans* LBJ in the bioremediation process to reduce the concentration of Pb in polluted soil, a leachate analysis was conducted for the bioaugmented sterile and non-sterile soils. A clear increase in the concentration of Pb in the leachate was observed, which proves that the bacteria *P*. *stutzeri* LBR and *C*. *metallidurans* LBJ raised the mobility and solubility of Pb in the soil. As a consequence, the bioavailability of this metal is increased, which required its transfer from the soil to the liquid fraction so that it can be easily precipitated and removed by the leaching process [33]. These results are consistent with the work of Reith and colleagues [42], who proved that microorganisms are the main drivers of heavy metal mobility. The bacteria *P*. *Stutzeri* LBR and *C*. *metallidurans* LBJ can therefore solubilize metals by production of siderophores and then adsorb metals on their biomass, on the external membrane proteins induced by the metals and by bioprecipitation. Of which, a relationship was observed between the bioavailability of heavy metals and the overproduction of siderophores [31].

## Conclusion

The results obtained in this work showed that microbial bioremediation with the strains *P*. *Stutzeri* LBR and *C*. *metallidurans* LBJ on the reduction of the concentration of Pb, in sterile and non-sterile polluted soil, can be considered as an effective method of environmental protection. A significant reduction in the concentration of Pb was particularly exhibited in the bioaugmented non-sterile (71.02%) and sterile (66.96%) soil in the presence of the consortium of both considered bacterial strains together. These two reduction rates in the concentration of Pb are still higher than that found in the soil bioaugmented with the bacterial strains *P*. *Stutzeri* LBR and *C*. *metallidurans* LBJ, separately. These results are confirmed by the analysis of sterile and non-sterile soil leachate, which proves an increase in the solubility, mobility and bioavailability of Pb from the solid fraction to the liquid fraction following an increase in the concentration of this metal in the leachate. In conclusion, the microbial bioremediation of polluted soil contaminated with Pb, by the strains *P*. *stutzeri* LBR and *C*. *metallidurans* LBJ, yielded very satisfactory results, hence the effectiveness of this eco-friendly biological process to clean up soils contaminated with heavy metals.

## Supporting information

S1 Table Cell growth of bacterial strains *P*. *stutzeri* LBR and *C*. *metallidurans* LBJ in sterile soil during bioaugmentation. (DOCX)

S2 Table Cell growth of bacterial strains *P*. *stutzeri* LBR and *C*. *metallidurans* LBJ in non-sterile soil during bioaugmentation. (DOCX)

S1 Fig Statistical data of days 0 of Pb concentration in sterile soil. (DOCX)

S2 Fig Statistical data of days 5 of Pb concentration in sterile soil. (DOCX)

S3 Fig Statistical data of days 10 of Pb concentration in sterile soil. (DOCX)

S4 Fig Statistical data of days 15 of Pb concentration in sterile soil. (DOCX)

S5 Fig Statistical data of days 20 of Pb concentration in sterile soil. (DOCX)

S6 Fig Statistical data of days 25 of Pb concentration in sterile soil. (DOCX)

S7 Fig Statistical data of days 0 of Pb concentration in sterile soil leachate. (DOCX)

S8 Fig Statistical data of days 5 of Pb concentration in sterile soil leachate. (DOCX)

S9 Fig Statistical data of days 10 of Pb concentration in sterile soil leachate. (DOCX)

S10 Fig Statistical data of days 15 of Pb concentration in sterile soil leachate. (DOCX)

S11 Fig Statistical data of days 20 of Pb concentration in sterile soil leachate. (DOCX)

S12 Fig Statistical data of days 25 of Pb concentration in sterile soil leachate. (DOCX)

S13 Fig Statistical data of days 0 of Pb concentration in non-sterile soil. (DOCX)

S14 Fig Statistical data of days 5 of Pb concentration in non-sterile soil. (DOCX)

S15 Fig Statistical data of days 10 of Pb concentration in non-sterile soil. (DOCX)

S16 Fig Statistical data of days 15 of Pb concentration in non-sterile soil. (DOCX)

S17 Fig Statistical data of days 20 of Pb concentration in non-sterile soil. (DOCX)

S18 Fig Statistical data of days 25 of Pb concentration in non-sterile soil. (DOCX)

S19 Fig Statistical data of days 0 of Pb concentration in non-sterile soil leachate. (DOCX)

S20 Fig Statistical data of days 5 of Pb concentration in non-sterile soil leachate. (DOCX)

S21 Fig Statistical data of days 10 of Pb concentration in non-sterile soil leachate. (DOCX)

S22 Fig Statistical data of days 15 of Pb concentration in non-sterile soil leachate. (DOCX)

S23 Fig Statistical data of days 20 of Pb concentration in non-sterile soil leachate. (DOCX)

S24 Fig Statistical data of days 25 of Pb concentration in non-sterile soil leachate. (DOCX)
