# Supplementary figures and images for: Bioremediation potential of consortium Pseudomonas Stutzeri LBR and Cupriavidus Metallidurans LBJ in soil polluted by lead
Source: PLoS One. 2023 Jun 15;18(6):e0284120. doi: 10.1371/journal.pone.0284120 (PMC10270627; doi:10.1371/journal.pone.0284120)

**
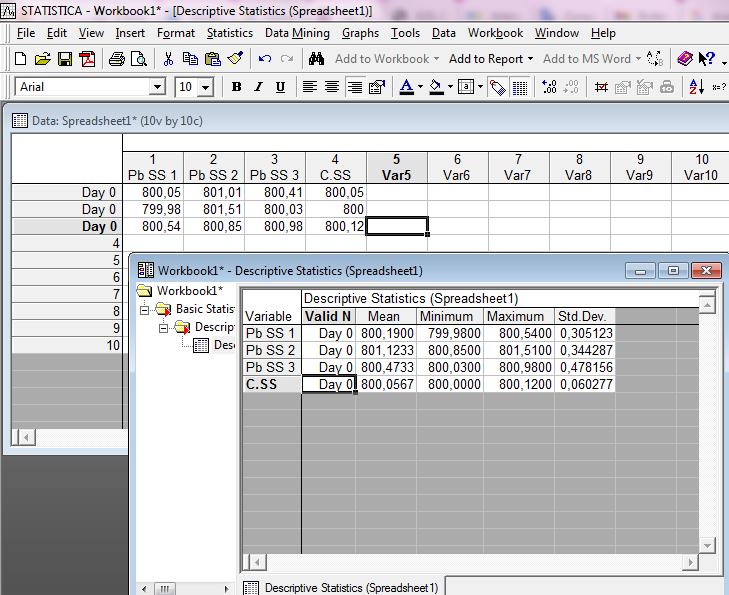
**

**S1 Fig. Statistical data of days 0 of Pb concentration in sterile soil**

Supplement: S1 Fig — (DOCX) [file pone.0284120.s003.docx]

**
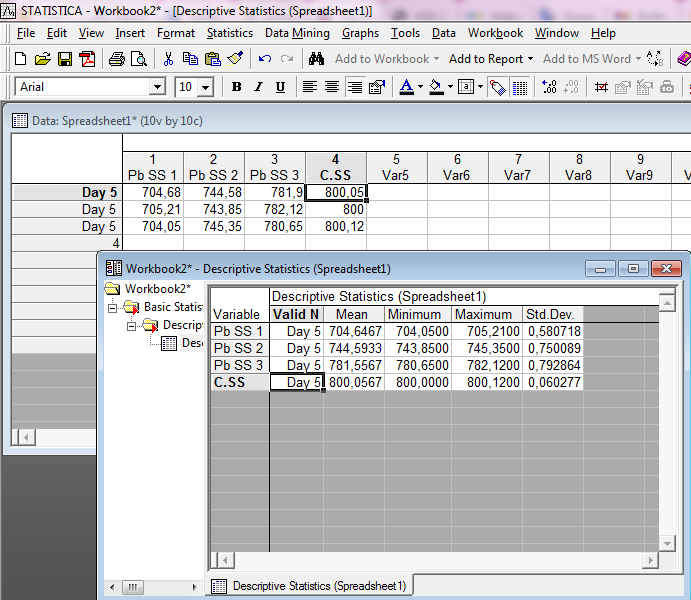
**

**S2 Fig. Statistical data of days 5 of Pb concentration in sterile soil**

Supplement: S2 Fig — (DOCX) [file pone.0284120.s004.docx]

**
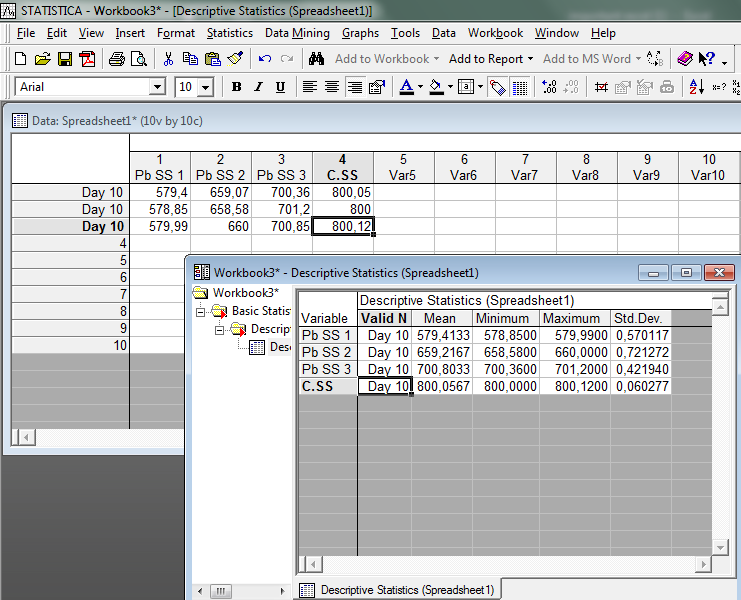
**

**S3 Fig. Statistical data of days 10 of Pb concentration in sterile soil**

Supplement: S3 Fig — (DOCX) [file pone.0284120.s005.docx]

**
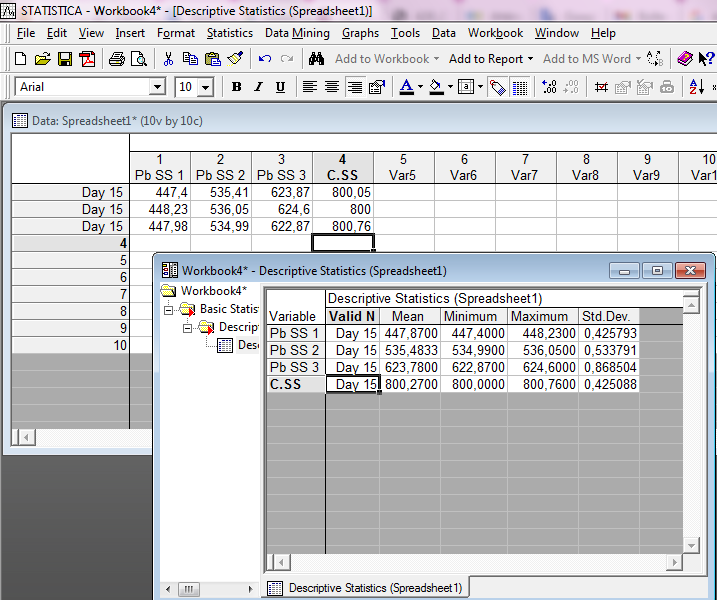
**

**S4 Fig. Statistical data of days 15 of Pb concentration in sterile soil**

Supplement: S4 Fig — (DOCX) [file pone.0284120.s006.docx]

**
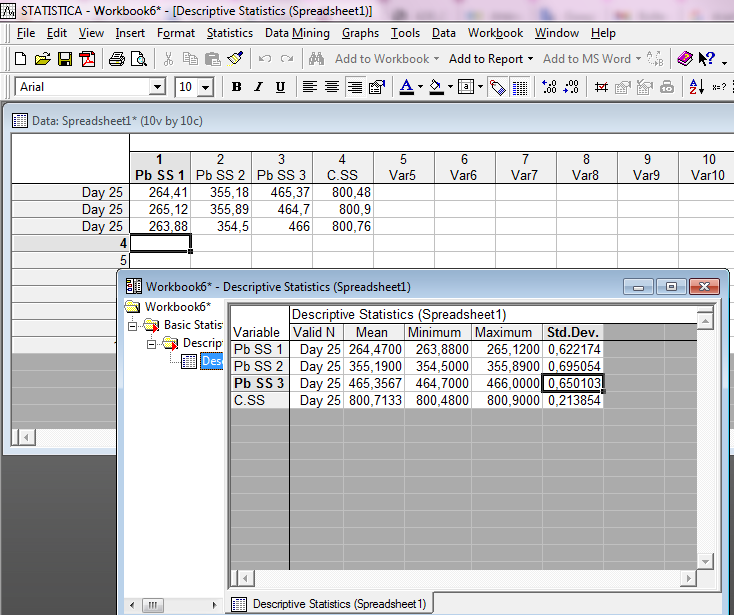
**

**S6 Fig. Statistical data of days 25 of Pb concentration in sterile soil**

Supplement: S6 Fig — (DOCX) [file pone.0284120.s008.docx]

**
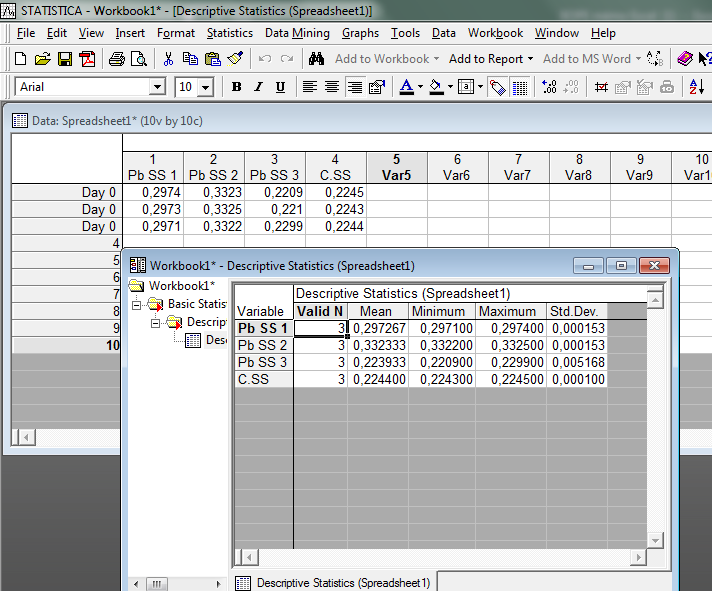
**

**S7 Fig. Statistical data of days 0 of Pb concentration in sterile soil leachate**

Supplement: S7 Fig — (DOCX) [file pone.0284120.s009.docx]

**
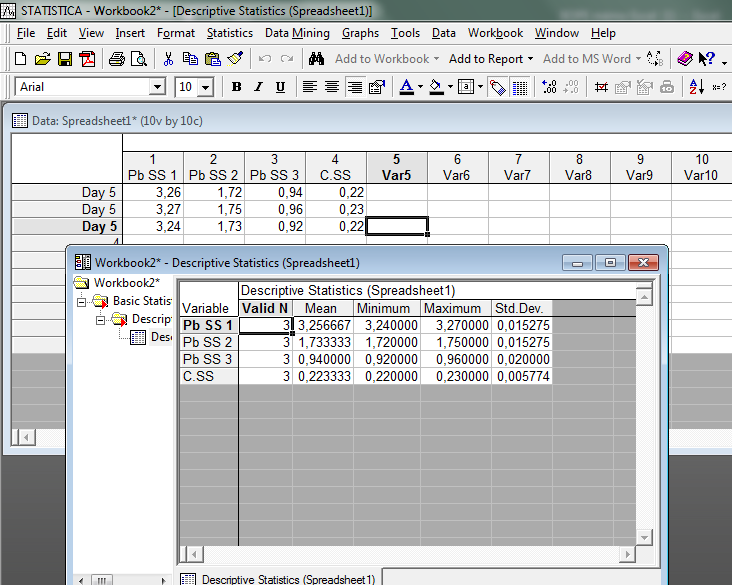
**

**S8 Fig. Statistical data of days 5 of Pb concentration in sterile soil leachate**

Supplement: S8 Fig — (DOCX) [file pone.0284120.s010.docx]

**
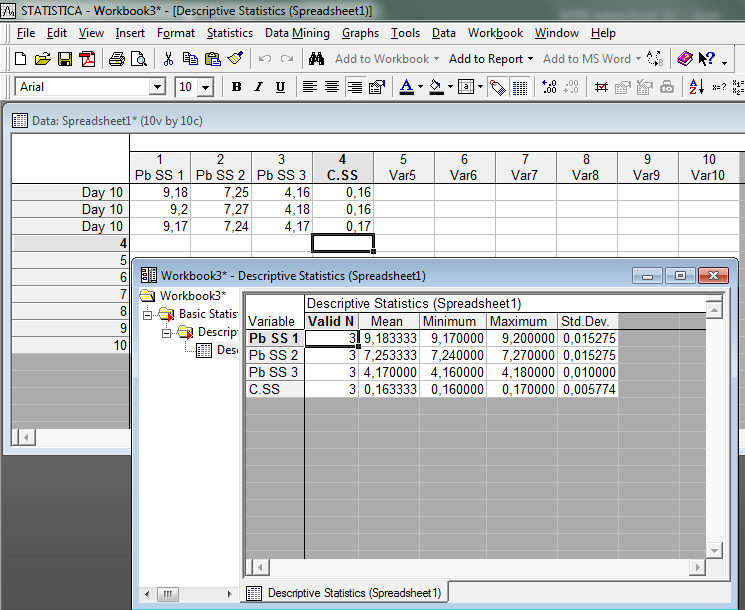
**

**S9 Fig. Statistical data of days 10 of Pb concentration in sterile soil leachate**

Supplement: S9 Fig — (DOCX) [file pone.0284120.s011.docx]

**
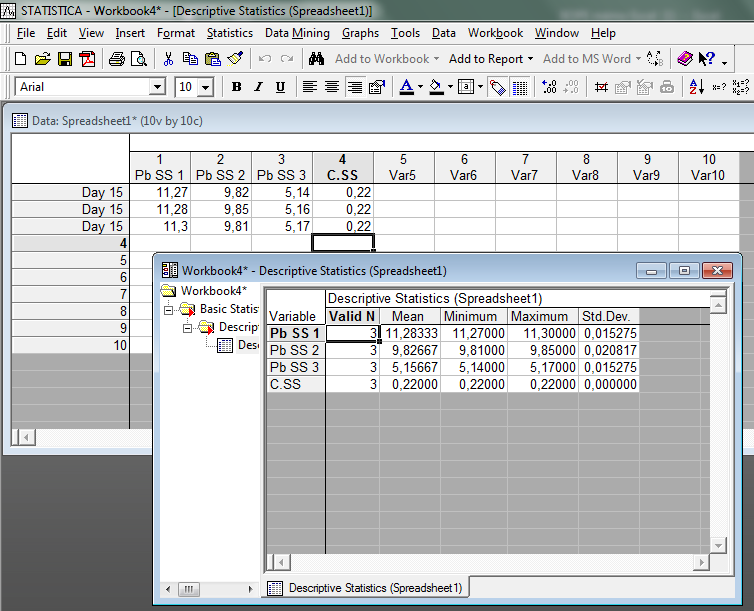
**

**S10 Fig. Statistical data of days 15 of Pb concentration in sterile soil leachate**

Supplement: S10 Fig — (DOCX) [file pone.0284120.s012.docx]

**
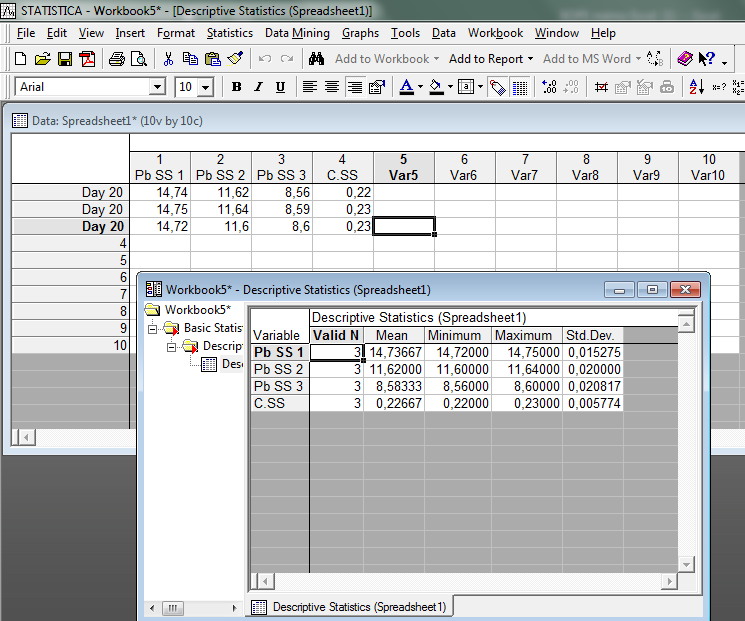
**

**S11 Fig. Statistical data of days 20 of Pb concentration in sterile soil leachate**

Supplement: S11 Fig — (DOCX) [file pone.0284120.s013.docx]

**
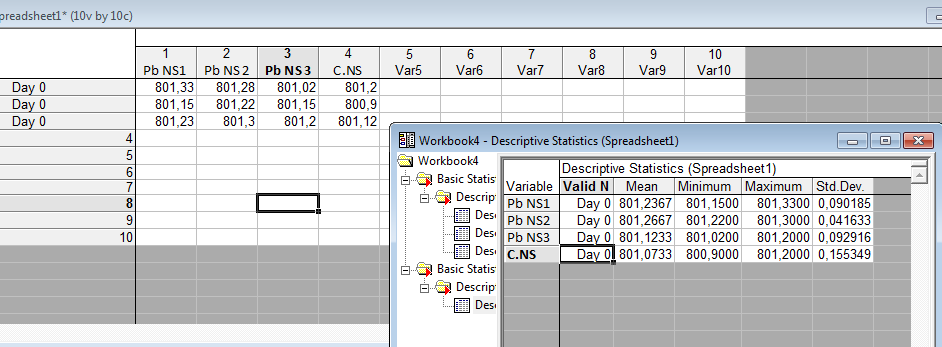
**

**S13 Fig. Statistical data of days 0 of Pb concentration in non-sterile soil**

Supplement: S13 Fig — (DOCX) [file pone.0284120.s015.docx]

**
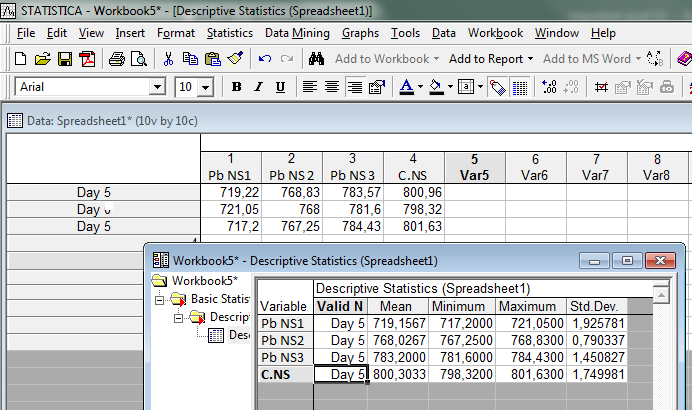
**

**S14 Fig. Statistical data of days 5 of Pb concentration in non-sterile soil**

Supplement: S14 Fig — (DOCX) [file pone.0284120.s016.docx]

**
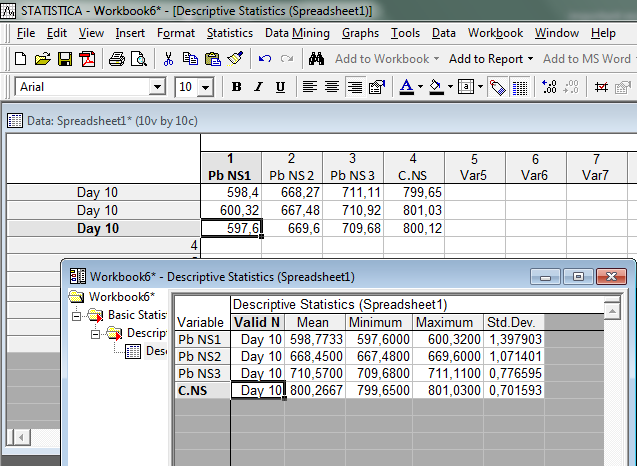
**

**S15 Fig. Statistical data of days 10 of Pb concentration in non-sterile soil**

Supplement: S15 Fig — (DOCX) [file pone.0284120.s017.docx]

**
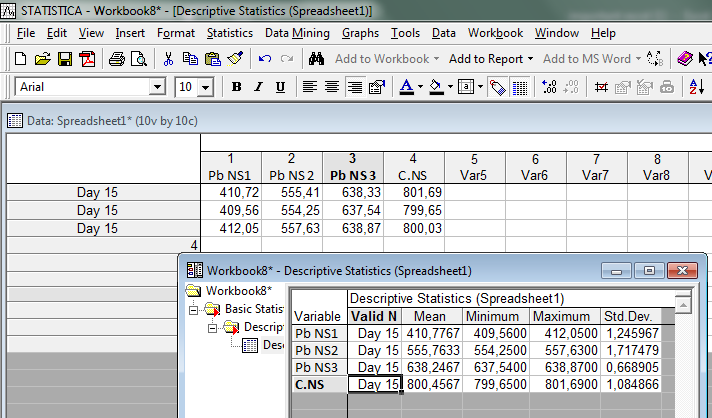
**

**S16 Fig. Statistical data of days 15 of Pb concentration in non-sterile soil**

Supplement: S16 Fig — (DOCX) [file pone.0284120.s018.docx]

**
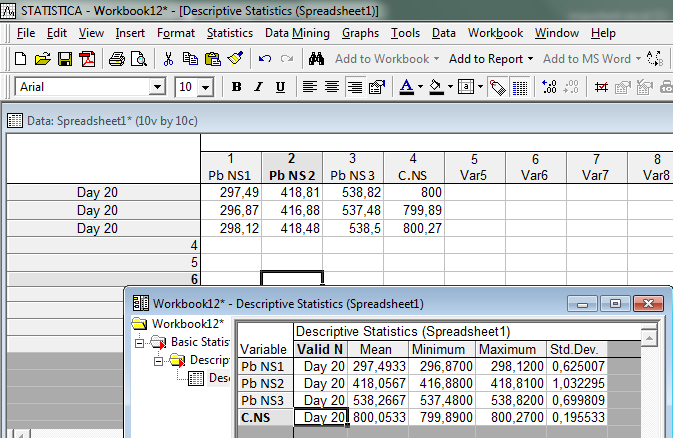
**

**S17 Fig. Statistical data of days 20 of Pb concentration in non-sterile soil**

Supplement: S17 Fig — (DOCX) [file pone.0284120.s019.docx]

**
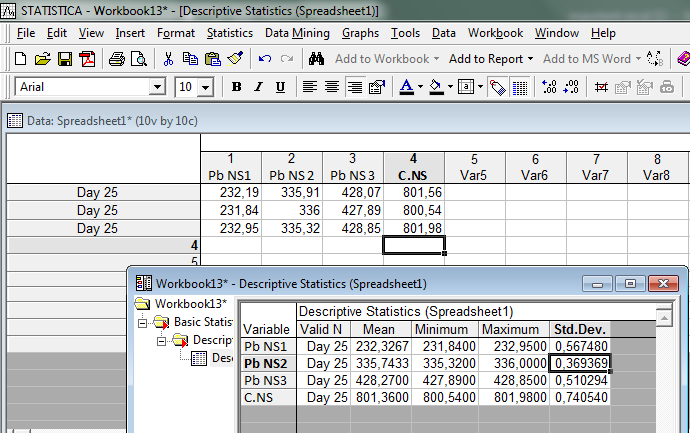
**

**S18 Fig. Statistical data of days 25 of Pb concentration in non-sterile soil**

Supplement: S18 Fig — (DOCX) [file pone.0284120.s020.docx]

**
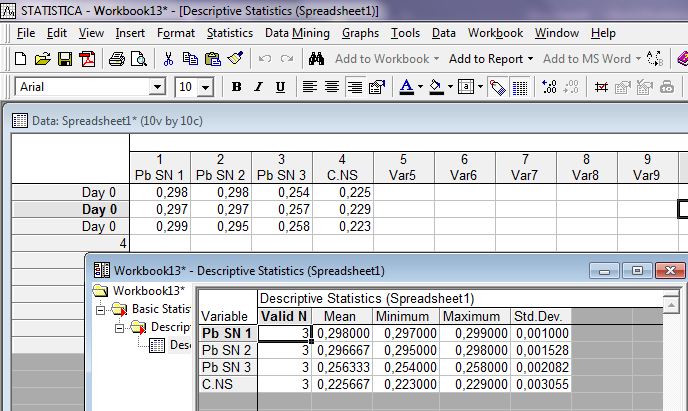
**

**S19 Fig. Statistical data of days 0 of Pb concentration in non-sterile soil leachate**

Supplement: S19 Fig — (DOCX) [file pone.0284120.s021.docx]

**
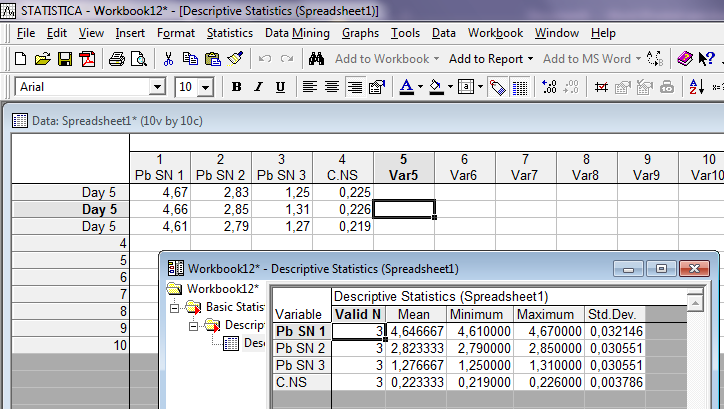
**

**S20 Fig. Statistical data of days 5 of Pb concentration in non-sterile soil leachate**

Supplement: S20 Fig — (DOCX) [file pone.0284120.s022.docx]

**
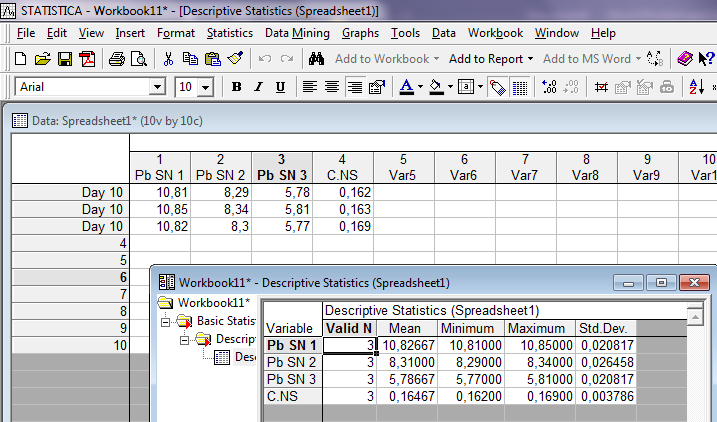
**

**S21 Fig. Statistical data of days 10 of Pb concentration in non-sterile soil leachate**

Supplement: S21 Fig — (DOCX) [file pone.0284120.s023.docx]

**
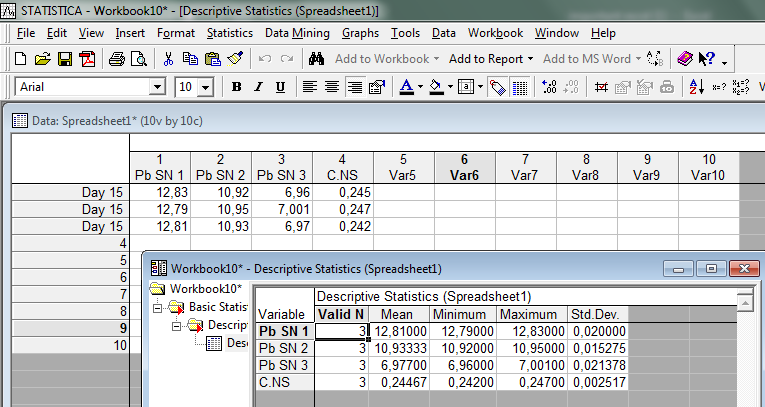
**

**S22 Fig. Statistical data of days 15 of Pb concentration in non-sterile soil leachate**

Supplement: S22 Fig — (DOCX) [file pone.0284120.s024.docx]

**
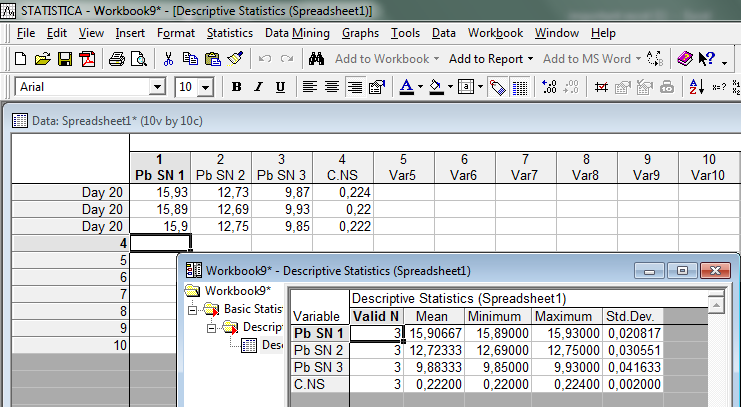
**

**S23 Fig. Statistical data of days 20 of Pb concentration in non-sterile soil leachate**

Supplement: S23 Fig — (DOCX) [file pone.0284120.s025.docx]

**
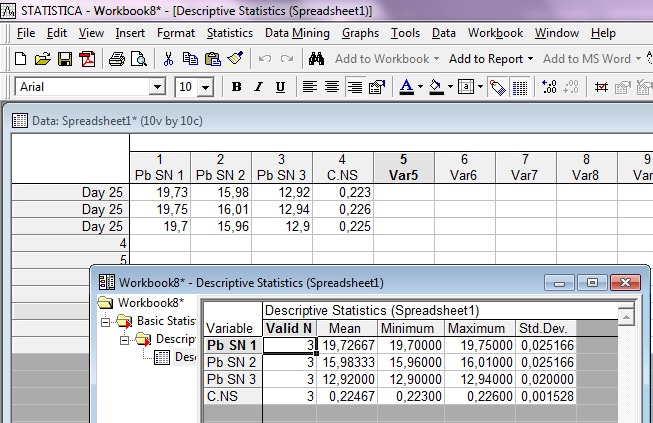
**

**S24 Fig. Statistical data of days 25 of Pb concentration in non-sterile soil leachate**

Supplement: S24 Fig — (DOCX) [file pone.0284120.s026.docx]
