# Supplementary material for: Bioremediation potential of consortium Pseudomonas Stutzeri LBR and Cupriavidus Metallidurans LBJ in soil polluted by lead
Source: PLoS One. 2023 Jun 15;18(6):e0284120. doi: 10.1371/journal.pone.0284120 (PMC10270627; doi:10.1371/journal.pone.0284120)
Supplement: S5 Fig — (DOCX) [file pone.0284120.s007.docx]

**Supporting Information**

**Table S1. Cell growth of bacterial strains *P. stutzeri* LBR and *C. metallidurans* LBJ in sterile soil during bioaugmentation**

| **Time (days)** | ***P. stutzeri* LBR + *C. metallidurans* LBJ**  **10^9^ CFU/mL** | ***P. stutzeri* LBR**  **10^9^ CFU/mL** | ***C. metallidurans* LBJ**  **10^9^ CFU/mL** |
| --- | --- | --- | --- |
| **Day 0** | 29 | 20 | 21 |
| **Day 5** | 35 | 26 | 25 |
| **Day 10** | 47 | 32 | 33 |
| **Day 15** | 65 | 53 | 45 |
| **Day 20** | 40 | 39 | 36 |
| **Day 25** | 25 | 22 | 18 |

**Table S2. Cell growth of bacterial strains *P. stutzeri* LBR and *C. metallidurans* LBJ in non-sterile soil during bioaugmentation**

| **Time (days)** | ***P. stutzeri* LBR + *C. metallidurans* LBJ**  **10^9^ CFU/mL** | ***P. stutzeri* LBR**  **10^9^ CFU/mL** | ***C. metallidurans* LBJ**  **10^9^ CFU/mL** |
| --- | --- | --- | --- |
| **Day 0** | 20 | 15 | 18 |
| **Day 5** | 32 | 28 | 25 |
| **Day 10** | 43 | 37 | 33 |
| **Day 15** | 58 | 52 | 46 |
| **Day 20** | 70 | 66 | 62 |
| **Day 25** | 65 | 60 | 55 |

**
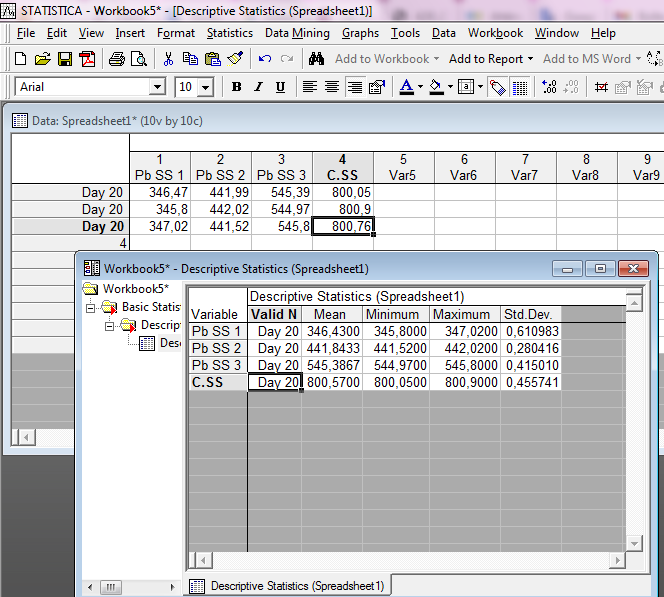
**

**S5 Fig. Statistical data of days 20 of Pb concentration in sterile soil**
