# Supplementary material for: Bioremediation potential of consortium Pseudomonas Stutzeri LBR and Cupriavidus Metallidurans LBJ in soil polluted by lead
Source: PLoS One. 2023 Jun 15;18(6):e0284120. doi: 10.1371/journal.pone.0284120 (PMC10270627; doi:10.1371/journal.pone.0284120)
Supplement: S12 Fig — (DOCX) [file pone.0284120.s014.docx]

**S12 Fig. Statistical data of days 25 of Pb concentration in sterile soil leachate**
